# Brain Training and Sulforaphane Intake Interventions Separately Improve Cognitive Performance in Healthy Older Adults, Whereas a Combination of These Interventions Does Not Have More Beneficial Effects: Evidence from a Randomized Controlled Trial

**DOI:** 10.3390/nu13020352

**Published:** 2021-01-25

**Authors:** Rui Nouchi, Qingqiang Hu, Toshiki Saito, Natasha Yuriko dos Santos Kawata, Haruka Nouchi, Ryuta Kawashima

**Affiliations:** 1 Department of Cognitive Health Science, Institute of Development, Aging and Cancer (IDAC), Tohoku University, Sendai 980-8575, Japan; haruka.nouchi.e8@tohoku.ac.jp; 2 Smart Aging Research Center (S.A.R.C.), Tohoku University, Seiryo-Machi 4-1, Sendai 980-8575, Japan; ryuta@tohoku.ac.jp; 3 Nature and Wellness Research Department, Innovation Division, Kagome Co., Ltd., 17, Nishitomiyama, Nasushiobara 329-2762, Japan; keikyo_ko@kagome.co.jp; 4 Department of Functional Brain Imaging, Institute of Development, Aging and Cancer (IDAC), Tohoku University, Sendai 980-8575, Japan; toshiki.saito@med.tohoku.ac.jp (T.S.); natashakawata@med.tohoku.ac.jp (N.Y.d.S.K.)

**Keywords:** sulforaphane, brain training, cognitive training, nutrition, multidomain intervention

## Abstract

Background: Earlier studies have demonstrated that a single-domain intervention, such as a brain-training (BT) game alone and a sulforaphane (SFN) intake, positively affects cognition. This study examined whether a combined BT and SFN intake intervention has beneficial effects on cognitive function in older adults. Methods: In a 12-week double-blinded randomized control trial, 144 older adults were randomly assigned to one of four groups: BT with SFN (BT-S), BT with placebo (BT-P), active control game (AT) with SFN (AT-S), and active control game with placebo (AT-P). We used Brain Age in BT and Tetris in AT. Participants were asked to play BT or AT for 15 min a day for 12 weeks while taking a supplement (SFN or placebo). We measured several cognitive functions before and after the intervention period. Results: The BT (BT-S and BT-P) groups showed more improvement in processing speed than the active control groups (AT-S and AT-P). The SFN intake (BT-S and AT-S) groups recorded significant improvements in processing speed and working memory performance unlike the placebo intake groups (BT-P and AT-P). However, we did not find any evidence of the combined intervention’s beneficial effects on cognition. Discussion: We discussed a mechanism to improve cognitive functions in the BT and SFN alone interventions.

## 1. Introduction

Cognitive functions decline with age [1] and are correlated with daily behaviors. For example, people with cognitive decline lose the ability to perform daily behaviors (e.g., bathing and shopping) [2]. Therefore, many researchers are interested in measures to improve cognitive functions. There are several intervention programs aimed at improving cognitive functions in older adults. Further, some meta-analysis and systematic review studies have reported that cognitive training [3] and nutrition intervention [4] have beneficial effects on the cognitive functions of older adults. In addition, recent studies indicate that a multidomain intervention improves a wide range of cognitive functions [5]. For example, a multidomain intervention including cognitive training and nutrition showed improvements in the cognitive functions of healthy older adults [6]. Therefore, combined cognitive training and nutrition interventions are expected to have more positive effects on cognitive function improvement than single-domain intervention programs in older adults. However, to date, there is no direct evidence of such benefits of a combined cognitive training and nutrition intervention. Therefore, this study investigated whether combined cognitive training and nutrition interventions have more beneficial effects on older adults’ cognitive functions than single-domain interventions.

Earlier studies on multidomain interventions have some limitations and present several unresolved issues. First, such studies usually used a multidomain intervention program that combined cognitive training, nutrition intervention, and exercise [5]. In addition, some other studies used a combination of nutrition [6,7] and exercise [8,9]. All these studies reported the beneficial effects of the combined cognitive training, nutrition, exercise interventions, and the combined nutrition and exercise intervention [8,9], as well. However, they did not clarify whether combined cognitive training and nutrition interventions have a positive effect on cognition. Second, earlier multidomain intervention studies used passive control groups that received usual care [7] or took health advice [6]. Such studies did not exclude the effect of social interactions or learning new things during an intervention program. In addition, to identify the beneficial effect of a multidomain intervention, it is better to set a single-domain intervention group as the comparison group. Third, earlier studies used a composite cognitive score that combined several cognitive test scores [6,7]. Although the use of such a composite score made it easy to analyze data and arrive at a simple result, it did not enable the identification of the cognitive domains that were improved by the multidomain intervention. To resolve these issues, we investigated whether the combined cognitive training with nutrition intake would have more beneficial effects on a wide range of cognitive functions in healthy older adults compared to a single-domain intervention (e.g., cognitive training alone or nutrition intervention alone).

This study considered a combination of a brain-training (BT) game and sulforaphane (SFN) intake. There are some reasons why we used Brain Age in cognitive training and sulforaphane intake as the nutrition intervention. First, Brain Age, which was developed by Nintendo, aims to train cognitive function through simple training games. The training games contain some simple mathematical calculations and prompts to read sentences aloud. Earlier studies have demonstrated that playing Brain Age leads to improvements in processing speed and executive functions in healthy young and older adults [10,11]. Second, SFN is an isothiocyanate found in cruciferous vegetables, such as cauliflower and broccoli. It has antioxidative and anti-inflammatory functions [12]. Further, a large cohort study reported that the amount of cruciferous vegetable intake was positively associated with memory performance, processing speed, and global cognitive functions in healthy older adults [13]. In addition, animal studies have reported that SFN intake leads to improvements in working memory performance [14]. Among human studies, one patient study revealed that SFN intake improved cognitive performance, including memory [15]. Therefore, SFN intake is expected to improve the cognitive functions of older adults.

Based on the evidence provided by earlier studies, we formulated three hypotheses. First, BT has a beneficial effect on processing speed and executive function performance, because earlier studies reported a medium-to-large effect of Brain Age on processing speed and executive function in healthy young and older adults [10,11,16]. Second, SFN intake leads to an improvement in working memory and processing speed performance, because earlier studies showed that SFN intake improved working memory performance in animals [14], and cruciferous vegetables have positive effects on processing speed in older adults [13]. Third, the combined BT and SFN intake intervention will improve multiple cognitive domains, unlike the single-domain intervention [6]. Overall, we expected that the combined BT and SFN intake intervention would have beneficial effects on a wider range of cognitive function performances compared to a single-domain intervention (i.e., BT alone, SFN intake alone, and active control groups). To validate these hypotheses, we conducted a randomized control trial (RCT) of the combined BT and SFN intake intervention in healthy older adults.

## 2. Materials and Methods

### 2.1. Randomized Controlled Trial Design and Setting

The RCT was conducted from July 2018 to July 2019 in Sendai city, Japan. The study protocol was approved by the Ethics Committee of the Tohoku University Graduate School of Medicine. Further, this RCT was registered at the University Hospital Medical Information Network Clinical Trial Registry (UMIN000032624). Trial registration: This trial was registered at The University Hospital Medical Information Network Clinical Trials Registry (UMIN 000032624). Registered 17 May 2018, https://upload.umin.ac.jp/cgi-open-bin/ctr/ctr_view.cgi?recptno=R000037199.

**Figure 1:**
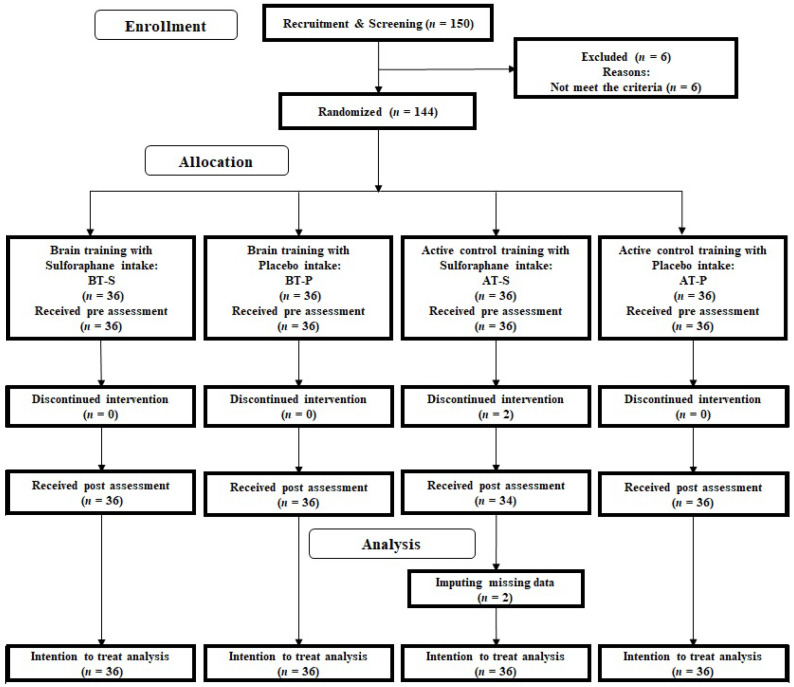
CONSORT diagram. BT: Brain training, AT: active control training, SFN: sulforaphane, P: placebo.

We conducted a double-blinded RCT on active control and placebo groups. Based on the 2 (cognitive training: BT and active control game (AT) groups) by 2 (nutrition: SFN and placebo groups) factor design, we considered BT with SFN (BT-S), BT with placebo (BT-P), AT with SFN (AT-S), and AT with placebo (AT-P) groups. All the participants and testers were blinded to the study hypotheses and the participants’ group membership. The primary outcome was cognitive function, and the secondary outcomes were emotional states. Further, the Consolidated Standards of Reporting Trials statement (http://www.consort-statement.org/home/, see Supplementary Table S1) was used to report the study structure. Figure 1 depicts the RCT design.

### 2.2. Participants

To recruit participants, we printed the inclusion and exclusion criteria on flyers in a local town paper. Subsequently, 150 individuals apprised the research group of their interest to participate over e-mail or by phone (Figure 1). We invited the respondents to attend an orientation meeting. During the meeting, one researcher (R.N.) explained the study details. After receiving informed consent from each participant, the researcher checked whether the interested participants were eligible to participate in the study. All the participants underwent a cognitive functional screening assessment using the Mini-Mental State Examination (MMSE) [17], Frontal Assessment Battery at bedside (FAB) [18], and Geriatric Depression Scale-15 (GDS) [19]. No participant was excluded based on their MMSE, FAB, or GDS scores. However, six participants who did not meet the inclusion criterion of medical history were excluded; subsequently, the remaining 144 participants were randomly assigned to four groups (BT-S, BT-P, AT-S, or AT-P). Two male participants in the AT-S group dropped out during the intervention period because of their inability to adhere to the study’s schedule. Based on the intention-to-treatment (ITT) analysis, we imputed the missing data using multiple imputation methods (Section 2.12). Table 1 depicts the baseline characteristics of all participants (*n* = 144; 48 men, 96 women; average age = 67.71 years (standard deviation, SD = 4.37)). There was no significant difference in baseline data among the groups.

### 2.3. Inclusion and Exclusion Criteria

Based on our earlier studies [10,20,21,22], we used the following inclusion criteria for this study: participants should (1) be right-handed; (2) be native Japanese speakers; (3) be 60–80 years of age; (4) not be using medications that are known to interfere with cognitive functions (including benzodiazepines, antidepressants, or other central nervous agents); (5) not have any history of diseases that are known to affect the central nervous system, including thyroid disease, multiple sclerosis, Parkinson’s disease, stroke, and diabetes; and (6) not have any food allergy concerns. The exclusion criteria were as follows: participants should (1) have an MMSE score less than 26, (2) have a FAB score less than 12, (3) have a GDS score more than 5, and (4) record the average amount of alcohol consumption to be more than 60 g per day. Finally, individuals who had participated in other cognition-related intervention studies were excluded, as well.

### 2.4. Sample Size Calculation

We calculated the sample size using the G*power software [23]. The sample size calculation was based on some studies using BT [10] and SFN [15]. For BT, earlier studies with 4-week intervention periods reported medium and large effect sizes (eta squired = 0.12 and 0.19 (vs. the active control group)) for processing speed in healthy older adults [10]. However, for SFN, no studies on healthy older adults have been recorded. One study on patients reported that 8 weeks of SFN intake improved multicomponent cognitive measurement, which included visual attention, processing speed, and memory, in patients with schizophrenia [15]. The study recorded a medium effect size (d = 0.77). Based on these studies, we expected a medium effect size (f = 0.24) in this study. To calculate the sample size, we used an analysis of covariance (ANCOVA) model with pre-processing speed score, MMSE, gender, and age as the covariates, using a two-tailed test, α = 0.05, and power = 0.80. Based on the 5% dropout ratio, the estimated sample size was 144 (36 participants in each group).

### 2.5. Randomization

To randomly assign the 144 interested participants to the four groups, we used an online randomization program (http://www.graphpad.com/quickcalcs/index.cfm). Further, we stratified the participants based on sex because earlier studies reported sex differences in cognitive function [24] as the primary outcome. We used blocked randomization (block size: 8) with an allocation ratio of 1:1:1:1.

### 2.6. Overview of the Intervention

Participants were asked to play a video game (either BT or AT) with the intake of supplements (SFN or placebo) at their homes for 15 min every day for 12 weeks. In this study, we used SFN and placebo supplements. The SFN supplements included 30 mg of the SFN precursor glucoraphanin, whereas the placebo supplements did not contain glucoraphanin. Both supplements had the same color and size. Therefore, the participants did not know which supplements they had consumed. We used a puzzle game (Tetris) for AT. This was the same protocol used in earlier studies [11]. The participants in both the groups had the same training period and a similar training setting, which reduced the effect of new experiences, such as performing cognitive tasks on a new device, and the effects of monitoring or maintaining the training schedule.

The participants played BT and AC training games at home on a portable video game console (Nintendo 2DS LL) having a video game software (Brain Age for BT and Tetris DS for AT) provided by the researchers. Prior to the intervention period, participants received instructions on how to use the console and play the training game. All of them played the training game on their own consoles. Further, the training duration was recorded by the console. Participants were asked to play the training games alone for approximately 15 min every day for 12 weeks. After finishing each training game, the participants were asked to write down all their game scores in a training note. At the end of the training period, they reported their subjective feelings of satisfaction and enjoyment with the training game on a five-point Likert scale: 1 = strongly disagree, 2 = disagree, 3 = neither agree nor disagree, 4 = agree, and 5 = strongly agree [25]. Further, cognitive tests and emotional state measurements were conducted before and after the 12-week intervention period. On the assessment day after the completion of the intervention period, the participants returned the console with the training game soft to the researchers.

### 2.7. Video Game Training

We used Brain Age published by Nintendo as the BT because many studies have demonstrated that playing Brain Age improved cognitive functions in healthy young and older adults [10,11]. This study used eight types of BT, as follows: “(1) In Calculation X 20, participants are required to answer a total of 20 simple arithmetic calculations as quickly as possible. The questions include addition, subtraction, and multiplication. (2) In Calculation X 100, participants are required to answer a total of 100 simple arithmetic calculations as in Calculation X 20. (3) In Reading Aloud, participants are required to read excerpts from Japanese classical literature aloud. (4) In Syllable Count, some sentences written in a combination of kanji and kana are presented. Participants are required to count the total number of kana letters after translating kanji sentences to kana. (5) In Low to High, numbers in boxes are first presented for several seconds. Then, participants are required to select the boxes from the lowest to the highest number. (6) In Head Count, participants watch scenes in which people enter or leave a house. Participants are then required to answer how many people are in the house at the end. (7) In Triangle Math, three numbers are presented on a top line (e.g., 5, 7, 2), two mathematical operations on a second line (e.g., +, +), and one mathematical operation (e.g., +) on the last line. Firstly, participants are required to solve the first formula (5 + 7) using the first two numbers (5, 7) in the first line and the first mathematical operation (+) in the second line, and then the second formula (7 + 2) using the last two numbers (7, 2) in the first line and the last mathematical operation (+) in the second line. Participants are then required to solve the last formula using the answer to the first formula (12), the answer to the last formula (9), and the mathematical operation (+) in the last line. In this case, participants give the final answer (21). (8) In Time Lapse, two analog clocks are presented. Participants are then required to calculate the difference in time between the two clocks. At the beginning of the game, participants can only perform three trainings (Calculation X 20, Calculation X 100, and Reading Aloud)” [10,11]. Every few days during the training, new training games were added to the game list. After playing the games, participants were asked to write down their game performances for each training game in a training book.

We used Tetris as the AT based on the findings of earlier studies [10,11]. Tetris is a popular block-puzzle game in which blocks drop from the top of the screen and participants can rotate and move the block and fit the blocks to make a complete line. Once a line is completed without any gaps, participants acquire game points. The aim of Tetris is to enable participants to acquire high game scores by forming complete lines. The active control group was designed to control the participants’ use of new devices and playing of video games and ensure that the intervention adheres to the fixed schedule.

### 2.8. Sulforaphane and Placebo Supplements

Participants received three capsules of the SFN or placebo supplements, which were prepared by Kagome Co., Ltd. (Nagoya, Japan), containing 30 mg or 0 mg of glucoraphanin per day, respectively, for 12 weeks. Glucoraphanin is known to be converted to SFN by gut microbial thioglucosidase [26]. The participants, tester, and researchers were blinded to the supplements until the completion of the study.

### 2.9. Cognitive Function Measurement

To examine the intervention’s effects on cognitive functions, we assessed participants’ game scores for processing speed, attention, inhibition, short-term memory, working memory, and episodic memory. Further, we measured the participants’ cognitive status using the MMSE, FAB, and Japanese Reading Test (JART). It took approximately 1.5 h for each participant to complete all the cognitive tests.

To obtain an overall assessment of the cognitive status, we used the MMSE, which measures individuals’ memory, attention, language, and visuospatial abilities [17]. Further, to ascertain participants’ reading ability and intelligence quotient, we used JART [27], which is a Japanese version of the National Adult Reading Test. JART is a reading test depicting 25 kanji (Chinese characters) compound words. The participants were asked to write the pronunciation of each kanji compound word.

To assess participants’ processing speed, we used digit symbol coding (Cd) and symbol search (SS) from the Wechsler Adult Intelligence Scale 3rd (WAIS-III) [28]. We reproduced the following descriptions of Cd and SS from our earlier report [29]: “For Cd, the participants were shown a series of symbols that were paired with numbers. Using a key within a 120-s time limit, participants drew each symbol under its corresponding number. The primary measure of this test was the number of correct answers. In SS, participants visually scanned two groups of symbols (a target group and a search group) and indicated whether either of the target symbols matched any symbol in the search group. Participants responded to as many items as possible within a 120-s time limit. The primary measure of this test was the number of correct answers.”

To measure participants’ attention performance, we conducted the digit cancellation task (D-CAT) and reproduced the following descriptions of D-CAT from our earlier report [11]: “The test sheet consists of 12 rows of 50 digits. Each row contains five sets of numbers 0–9 arranged in a random order. Thus, any one digit appears five times in each row with randomly determined neighbors. The D-CAT consists of three such sheets. Participants were instructed to search for the target number(s) that had been specified to them and to delete each one with a slash mark as quickly and as accurately as possible until the experimenter sent a stop signal. Three trials were conducted, first with a single target number (6), second with two target numbers (9 and 4), and third with three numbers (8, 3, and 7). Each trial was given 1 min. Consequently, the total time required for D-CAT was 3 min. For the second and third trials, it was emphasized that all the instructed target numbers should be canceled without omission. The primary measure of this test was the number of hits (correct answers). We used only the number of hits in the first trial.”

To measure the inhibition ability of executive functions, we used a Stroop task (ST) and a reverse Stroop task (rST) [30], as follows: “In the ST, in the leftmost of six columns, a word naming a color was printed in another color (e.g., ‘red’ was printed in blue letters); the other five columns contained words naming colors. Participants were required to check the column containing the word naming the color of the word in the leftmost column. In the rST, in the leftmost of six columns, a word naming a color was printed in another color (e.g., ‘red’ was printed in blue letters); the other five columns were filled with five different colors, from which participants were required to check the column with the color matching the word written in the leftmost column. In each task, participants were instructed to complete as many of these exercises as possible in 1 min. The primary measure for this task was the number of correct items” [29].

To measure short-term memory and working memory performance, we used the digit span forward (DS-F) and digit span backward (DS-B) tasks, which are the subtests of WAIS-III [28]. We reproduced the following descriptions of DS-F and DS-B from our earlier report [29]: “For the DS-F, participants repeated numbers in the same order as they were read aloud by the examiner. For the DS-B, participants repeated numbers in the reverse order of that read aloud by the examiner. In both the tasks, the examiner read a series of number sequences which the participant was required to repeat in either the forward or reverse order.” The primary measure of this test was the digit number length. The maximum digit number length in DS-F was 8 and that in DS-B was 7.

To measure episodic memory, we used the logical memory (LM) subtest of the Wechsler Memory Scale-Revised (WMS-R) [31], which is described as follows: “LM consists of two short-paragraph-length stories (Story A and Story B). For the LM, participants were required to memorize one of the two stories. The stories were scored in terms of the number of story units recalled, as specified in the WMS-R scoring protocol. We used either Story A or Story B. The primary measure for this task was the number of correct story units recalled” [29]. In this manner, we checked immediate recall and delayed recall memory performances.

To measure visuospatial ability, we used a mental rotation (MR) test [32]. The MR test uses three-dimensional cubical figures and has 24 items. Each item comprises a row of five drawings, with a target figure in the leftmost position followed by four response-choice figures. The participants were asked to find the two choice figures that were the rotated reproductions of the target figure.

### 2.10. Emotional State Measurement

To assess the change in a participant’s emotional state, we used a short version of the Profile of Mood State Second Edition (POMS2) [33,34]. In the POMS2, 35 items are rated on a 5-point scale Likert scale (from 1 to 5). The POMS2 can measure the following emotional states in the prior week: tension–anxiety (T–A), depression–dejection (D), anger–hostility (A–H), vigor–activity (V), fatigue–inertia (F–I), confusion–bewilderment (I), and friendliness (F). In the POMS2, we calculated the participants’ total mood disturbance (TMD) scores using six subscores.

The World Health Organization-Five Well-Being Index (WHO-5) is a measure of subjective mental well-being within two weeks [35]. The WHO-5 has five items with 6-point scales (with responses ranging from 0 to 5). A high score indicates good mental well-being.

The General Health Questionnaire (GHQ) is widely used to detect common psychiatric disorders. In this study, we used the GHQ with 12 items (GHQ12) [36]. Each item assessed the severity of a mental problem for the past few weeks using a 4-point Likert-type scale (responses ranging from 0 to 3).

Finally, we used the Center for Epidemiologic Studies Depression Scale (CES-D) to measure depressive symptoms [35]. The CES-D has 20 items. Participants subjectively rated their feelings and behaviors during the past week on 4-point scales (with responses ranging from 0 to 3).

### 2.11. Urine Analysis

To assess whether SFN was absorbed into the body, the urine excretion level of sulforaphane N-acetyl-L-cysteine (SFN-NAC), a major metabolite of SFN, was analyzed in urine samples collected from the participants 6–12 h after their final intake of the SFN or placebo supplement. Urine samples (stored at −30 °C) were thawed on ice and centrifuged (14,000× *g*, 15 min, 4 °C) to precipitate proteins. The supernatants were filtered through Ultrafree^®^-MC, GV 0.22-μm centrifugal filters (Merck Millipore, Bedford, MA, USA), and the filtrates (25 μL) along with the internal standard iberin were subjected to HPLC–MS/MS analysis (a Shimadzu 20A HPLC system (Shimadzu, Kyoto, Japan) coupled to an LCQ Fleet electrospray ionization (ESI) ion trap mass spectrometer (Thermo Scientific, San Jose, CA, USA) in duplicate). The LC conditions involved a slight modification of Egner’s method [37] and were as follows: column, COSMOSIL 5C18-AR-II column (2.1 × 100 mm, Nacalai Tesque, Kyoto, Japan); mobile phase (*v*/*v*/*v*), (A) water/acetonitrile/acetic acid (95/5/0.1) and (B) acetonitrile/acetic acid (100/0.1) with a linear elution gradient of 0%–60% B over 15 min at 0.2 mL/min at 40 °C. The MS/MS measurement condition for a time segment definition of 10.5–14.5 min in an ESI-positive ionization mode was as follows: Sheath gas flow rate, 16 psi; aux gas flow rate, 0 psi; scan ranges, 50–500 *m*/*z*; capillary temperature, 275 °C; capillary voltage, 48 V; tube lens, 100 V; radiofrequency (RF) lens offset −6.5 V; lens 0 voltage, −4.0 V; lens 1 voltage, −9.0 V; multipole 0 offset, −5.0 V; multipole 1 offset, −8.5 V; and multipole RF amplitude, 400.0 V. Further, the monoisotopic isolations (*m*/*z*) for SFN-NAC and iberin at a width of *m*/*z* 1.0 were 340.0 > 178.1 and 164.0 > 105.1, respectively. The quantitation of SFN-NAC was based on a 5-point standard curve, and the internal standard was dependent on the presence of two peaks monitored with the expected area ratio. Finally, the urinary levels of SFN-NAC were standardized to creatinine levels, which were determined using a commercial kit (Exocell, Philadelphia, PA, USA) according to the manufacturer’s instructions.

### 2.12. Analyses

All analyses were performed using the software R (ver. 3.53). All participants were included in the analyses according to the ITT principle. First, we imputed missing data using the multiple imputation method (predictive mean matching, m = 20). All variables associated with the participants’ pre-scores, post-scores, and basic information (age, sex, MMSE score, and FAB score) were included in the data imputation process. We performed multiple imputations using the “mice” function in the mice package of R [38]. Subsequently, we calculated the changes in participants’ cognitive function and emotional state scores (the post-intervention score minus pre-intervention score). Then, using all the 20 imputed datasets, we performed a 2 (video game: BT or AT) by 2 (intake supplement: SFN or placebo) ANCOVA with permutation tests for all the change scores. We performed permutation tests because they are suitable for small-sample analyses and are distributed freely. In the ANCOVA, the change score was the dependent variable, and the video game factor (BT or AT) and supplement factor (SFN or placebo) were independent variables. The pre-scores for the dependent variable, MMSE score, age, and sex, were used as covariates. All the ANCOVAs with permutation tests were performed using the “aovp” function in the lmPerm package (http://cran.r-project.org/web/packages/lmPerm/index.html). Finally, we used false discovery rate correction methods to adjust all the pooled *p* values [39]. The value *p* < 0.05 was considered significant for multiple comparison methods. In addition, to confirm that SFN was absorbed into the participants’ bodies, we conducted a two-sample *t*-test (SFN vs. placebo) for SFN-NAC urinary levels.

## 3. Results

In this study, we checked participants’ adherence to playing video games and the intake of supplements using a 2 (video game: BT or AT) by 2 (intake supplement: SFN or placebo) ANCOVA. The analysis did not reveal any significant main effects (*p* > 0.05) or interaction effects (*p* > 0.05) on the average number of training days (maximum = 84 days: BT-S: *Mean* = 83.56 (*SD* = 0.65), BT-P: *Mean* = 83.22 (*SD* = 1.14), AT-S: *Mean* = 83.76 (*SD* = 1.33), and AT-P: *Mean* = 83.17 (*SD* = 1.47)) and number of supplement-intake days (maximum = 84 days: BT-S: *Mean* = 83.31 (*SD* = 2.08), BT-P: *Mean* = 82.03 (*SD* = 1.64), AT-S: *Mean* = 82.79 (*SD* = 1.81), and AT-P: *Mean* = 82.56 (*SD* = 1.78)). Further, we evaluated participants’ satisfaction and enjoyment after the intervention using a five-point scale. There was no significant difference in the average enjoyment score (BT-S: *Mean* = 5.17 (*SD* = 1.03), BT-P: *Mean* = 5.17 (*SD* = 1.01), AT-S: *Mean* = 5.28 (*SD* = 1.26), and AT-P: *Mean* = 5.33 (*SD* = 1.08)), and satisfaction score (BT-S: *Mean* = 4.03 (*SD* = 1.42), BT-P: *Mean* = 4.17 (*SD* = 1.33), AT-S: *Mean* = 3.88 (*SD* = 1.29), and AT-P: *Mean* = 4.33 (*SD* = 1.20)) among the groups. Finally, there was no significant difference among the groups in terms of the baseline measures (Table 1).

Two participants in the AT-S group dropped out of the study during the intervention period because they could not adhere to the study schedule. We analyzed the data using the ITT rule and imputed missing data (Section 2.12). Further, we performed a permutation test with 2 (cognitive training: BT or AT) by 2 (nutrition: SFN or placebo) ANCOVAs on the change scores (Table 2 and Table 3). We found a significant main effect of cognitive training on processing speed performance (SS: *F* (1, 135) = 7.054, *η*^2^ = 0.06, *adjusted p* < 0.05, Cd: *F* (1, 135) = 7.743, *η*^2^ = 0.07, *adjusted p* < 0.05) and a significant main effect of nutrition on processing speed (SS: *F* (1, 135) = 9.05, *η*^2^ = 0.08, *adjusted p* < 0.05) and working memory (BS: *F* (1, 135) = 8.44, *η*^2^ = 0.07, *adjusted p* < 0.05) performances. However, we did not find any significant main effects or interaction effects of cognitive training or nutrition on cognitive performance or emotional state (Table 4). In summary, the BT groups demonstrated improvements in processing speed compared to the AT groups. Finally, the SFN intake group demonstrated improvements in processing speed and working memory performance compared to the placebo intake group.

In addition, urinary SFN-NAC was confirmed to be significantly higher in the SFN intake group (*Mean* = *4.2*, SD = 6.1) than in the placebo intake group (*Mean* = 1.8, *SD* = 4.1; *t* (140) = 2.71, *p* = 0.008, *d* = 0.45). To investigate the relationships among these improved performances and urinary SFN-NAC, we performed multiple regression analyses. The covariates were age, sex, baseline MMSE score, and the pre-score on the dependent variable. We found no significant correlation between improved cognitive function and urinary SFN-NAC.

## 4. Discussion

This study investigated whether the combined BT and SFN intake intervention improved a wide range of cognitive function performances in healthy older adults. We did not find any evidence to support the combined intervention’s beneficial effects on a wide range of cognitive functions. However, two main findings of the study support our hypotheses related to the effects of BT and SFN intake. The first finding is that the BT group showed a significant improvement in processing speed measured using SS and Cd compared to the active control group. The second finding is that the SFN intake group demonstrated significant improvement in processing speed, which was measured using SS, and working memory performance, which was measured using DS-B, compared to the placebo intake group. In this section, we discuss these findings separately.

The first finding is that BT improved processing speed measured using SS and Cd. This result supports our hypothesis. Further, it is consistent with the findings of earlier studies on young and older adults [10,11]. Whereas earlier research [10,11] considered a short intervention period (4 weeks) and small-sample studies (less than 40 participants), the current study expanded an earlier finding to reveal an improvement in the processing speed of healthy older adults by considering a longer intervention period (12 weeks). On the other hand, we did not identify any improvements in executive function measured using ST and rST in the BT group. An earlier study using the same BT showed significant improvements in executive functions measured by the FAB and the trail making test B after 4 weeks of intervention in healthy older adults [10]. In addition, some other studies reported improvements in the executive function measured using ST and rST after a 4-week intervention period in healthy young adults [11,16]. Further, ST performance improved after 8 weeks of BT in multiple sclerosis patients [40,41]. However, there are some methodological differences (in terms of cognitive measures, intervention period, and participants) between the current study and the aforementioned earlier studies. Hence, it is difficult to conclude whether BT has any beneficial effects on executive functions. Future studies should examine whether a 12-week BT intervention leads to improvements in executive functions using multiple executive functional tests, including the ST and trail making test.

SS and Cd improvements in processing speed after BT are explained by the overlapping hypothesis [16,25]. According to the overlapping hypothesis, cognitive performance can undergo change after training when trained and untrained measures (e.g., cognitive performance tests) share common mental and behavioral processes. The overlapping hypothesis is based on the Cattel–Horn–Carroll (CHC) model [42]. In the CHC model, behavioral and mental processes are divided into three levels: narrow, broad, and general abilities. For BT, calculation and reading, which are the main components of BT, are general abilities that include several narrow and broad abilities. In this study, we requested participants to complete the training games as quickly as possible. Therefore, BT requires the demonstration of a broad ability, such as processing speed. The overlapping hypothesis further explains the improvement in processing speed performance in the following manner: In BT, calculation and reading training mainly require processing speed as a mental process. Playing BT is expected to facilitate and enhance processing speed. Therefore, BT directly improved participants’ processing speed because the mathematical calculation and reading training included in BT required the participants to demonstrate their processing speed.

The second finding is that SFN intake improved the SS performance in processing speed and DS-B in working memory. This result is in line with previous evidence. For example, an animal study reported that SFN intake enhanced working memory performance [14]. Further, one patient study showed that 8 weeks of SFN intake improved the multicomponent cognitive measurement, which included visual attention, processing speed, and memory, of patients with schizophrenia [15]. In addition, a cohort study revealed a positive correlation between the amount of cruciferous vegetable intake and processing speed [13]. However, this study is the first to provide direct evidence that SFN intake improves processing speed and working memory performance in healthy older adults. Further, these findings support our hypothesis related to the effect of SFN intake.

The antioxidative and anti-inflammatory properties of SFN explain its ability to improve processing speed and working memory. Further, inflammation and oxidative stress levels were negatively correlated with general cognitive functions in older adults [43]. An earlier study indicated that the antioxidant and anti-inflammatory properties of different types of food play an important role in maintaining and improving cognitive functions [44]. It is noted that SFN has antioxidative and anti-inflammatory functions [12]. Further, some animal studies demonstrated that SFN improved behavioral performance, including behavior speed and working memory performance [14,45]. In addition, one human patient study reported that SFN intake led to an improvement in multicomponent cognitive functions [15]. The antioxidant and the anti-inflammatory properties of SFN play a critical role in improving cognitive functions [46]. Taken together, the 12-week SFN intake reduced inflammation and oxidative stress levels. Further, the anti-inflammatory and antioxidative functions lead to improvements in processing speed and working memory performance in older adults. However, the current study did not measure the antioxidant and anti-inflammatory properties of SFN. Therefore, to confirm our hypothesis, future studies should measure the antioxidant and anti-inflammatory properties of SFN.

Further, the combined BT and SFN intervention did not improve multiple cognitive performances compared to separate BT and SFN intake interventions. Earlier studies reported that a 2- or 3-year long-term multidomain intervention program, which included cognitive training, exercise, and nutrition, had positive effects on multiple cognitive performances and composite cognitive score of healthy older adults [6,7]. One possible reason why we did not find any improvements in a wide range of cognitive domains is the short intervention period. For example, the current study considered an intervention period of 12 weeks. This was shorter than the intervention periods (2 or 3 years) considered by studies using multidomain interventions [6,7]. In this study, the 12-week intervention period was not sufficient to detect the multidomain intervention’s beneficial effects on a wide range of cognitive domains. Therefore, future studies should consider longer intervention periods to examine whether a combined cognitive training and SFN intake intervention affects a wide range of cognitive domains.

This study has some limitations. First, we did not find any significant correlation between urinary SFN-NAC and improvements in cognitive performance. However, urinary SFN-NAC is only one indicator of whether SFN is absorbed into the body or not. We found significant differences in the urinary SFN-NAC values between the SFN intake and placebo groups, which implies that the SFN intake increased the absorbed SFN level. On the other hand, we did not find any significant correlation between cognitive improvement and urinary SFN-NAC. There are some possible reasons for this finding (e.g., a small sample size, a narrow range of SFN-NAC, and the spot measurement of urinary SFN-NAC). Therefore, future studies should investigate the association between the absorption of SFN and the improvements in cognitive performance using other methods, such as blood sampling. Second, we did not consider details regarding the dietary patterns of participants. Individual differences in dietary pattern at baseline may influence the effects of SFN intake on cognitive functions. For example, SFN supplements may not benefit people who regularly consume broccoli sprouts. Therefore, it is important that future studies consider the dietary patterns of participants. Third, we did not conduct any follow-up assessments. We measured participants’ cognitive functions immediately after the intervention period and ignored the possibility that the combined BT and SFN intake intervention may have long-term effects on cognition. Therefore, it is important to investigate the duration of the beneficial effects of the combined BT and SFN intake.

## 5. Conclusions

We investigated the effects of a 12-week-long combined BT and SFN intake intervention on cognitive function in healthy older adults. Although we did not find any evidence to support the intervention’s beneficial effects on cognitive functions, we found that the BT and SFN intake separately led to improvements in cognitive functions. The BT group showed a significant improvement in processing speed compared to the active intervention group. Further, the SFN intake groups revealed significant improvements in processing and working memory performance in the placebo supplement intake group.

## Figures and Tables

**Table 1:** Age, depression, and general cognitive function scores of the participant groups at baseline.

Cognitive Training	BT		AT	
Nutrition	SFN	P	SFN	P
Age (years)	67.97	67.42	67.59	67.86
	(3.12)	(4.78)	(4.58)	(4.92)
Sex	Male = 12 Female = 24	Male = 12 Female = 24	Male = 12 Female = 24	Male = 12 Female = 24
MMSE	28.36	28.42	28.50	28.56
	(1.29)	(1.44)	(1.29)	(1.21)
FAB	14.53	14.72	14.18	14.25
	(1.73)	(1.67)	(1.75)	(1.75)
JART	19.44	19.22	20.71	20.19
	(3.64)	(4.75)	(3.28)	(3.55)
GDS	3.12	3.45	3.31	3.21
	(1.04)	(1.21)	(0.91)	(1.24)
WHO-5	15.75	16.39	15.44	16.56
	(4.59)	(4.67)	(4.31)	(3.04)
GHQ12	21.92	22.58	21.24	21.19
	(4.28)	(4.74)	(3.69)	(3.85)
POMS	21.31	22.92	24.09	22.75
	(12.47)	(10.42)	(12.05)	(12.62)
CES-D	11.06	11.08	9.68	9.64
	(7.58)	(5.74)	(6.34)	(5.14)

Note: BT: Brain training, AT: active control training, SFN: sulforaphane, P: placebo, MMSE: Mini-Mental State Examination, FAB: Frontal Assessment Battery at bedside, JART: Japanese Reading Test, GDS: Geriatric Depression Scale, WHO-5: World Health Organization-Five Well-Being Index, GHQ12: General Health Questionnaire (GHQ) with 12 items, POMS: total mood disturbance (TMD) score in the Profile of Mood State, CES-D: Center for Epidemiologic Studies Depression Scale.

**Table 2:** Cognitive functions of the participant groups at baseline.

Cognitive Training	BT		AT	
Nutrition	SFN	P	SFN	P
LM-immediate	10.78	10.78	10.53	11.69
	(4.57)	(3.60)	(3.42)	(4.63)
LM-delayed	10.44	9.69	9.65	10.86
	(4.36)	(3.55)	(3.27)	(4.54)
Cd	72.03	72.81	75.24	72.89
	(13.48)	(12.27)	(15.81)	(12.46)
SS	34.39	37.19	36.29	36.44
	(4.96)	(6.04)	(6.53)	(6.72)
D-CAT	45.78	46.44	47.21	47.03
	(7.30)	(12.13)	(11.51)	(9.71)
rST	45.47	46.97	49.32	45.86
	(9.07)	(7.69)	(7.21)	(7.54)
ST	32.67	31.97	32.85	33.67
	(8.71)	(7.82)	(8.36)	(7.94)
DS-F	5.67	5.56	5.38	5.64
	(0.83)	(1.05)	(1.02)	(1.07)
DS-B	4.25	4.31	4.32	4.47
	(0.94)	(0.92)	(1.20)	(1.11)
MR	18.36	16.83	18.06	17.83
	(4.65)	(6.10)	(5.83)	(4.66)

Note: BT: Brain training, AT: active control training, SFN: sulforaphane, P: placebo, LM: logical memory, Cd: digit symbol coding, SS: symbol search, D-CAT: digit cancelation task, rST: reverse Stroop task, ST: Stroop task, DS-F: digit span forward, DS-B: digit span backward, MR: mental rotation.

**Table 3:** Change scores of cognitive functions in all the participant groups.

Cognitive Training	BT		AT	
Nutrition	SFN	P	SFN	P
LM-immediate	−0.33	−0.33	0.19	−0.89
	(3.46)	(2.61)	(4.01)	(3.67)
LM-delayed	0.39	0.64	0.97	−0.39
	(3.40)	(2.74)	(3.64)	(3.16)
Cd	4.94	2.78	2.42	1.33
	(4.88)	(5.25)	(6.54)	(4.54)
SS	3.47	1.39	1.47	0.08
	(2.95)	(3.50)	(3.80)	(4.12)
D-CAT	0.67	−2.97	6.56	10.53
	(21.66)	(24.49)	(29.28)	(18.34)
rST	0.81	−0.06	0.64	0.67
	(4.57)	(4.90)	(4.40)	(4.08)
ST	−0.11	0.67	−0.81	1.00
	(3.73)	(3.49)	(7.09)	(5.54)
DS-F	0.31	0.11	0.44	0.56
	(1.45)	(1.53)	(1.50)	(1.42)
DS-B	0.92	0.36	0.81	−0.11
	(1.56)	(1.50)	(1.55)	(1.75)
MR	1.06	0.56	1.06	0.08
	(5.36)	(4.31)	(5.39)	(3.62)

Note: BT: Brain training, AT: active control training, SFN: sulforaphane, P: placebo, LM: logical memory, Cd: digit symbol coding, SS: symbol search, D-CAT: digit cancelation task, rST: reverse Stroop task, ST: Stroop task, DS-F: digit span forward, DS-B: digit span backward, MR: mental rotation.

**Table 4:** Change scores of emotional states in all groups.

Cognitive Training	BT		AT	
Nutrition	SFN	P	SFN	P
WHO-5	−0.19	0.03	−0.24	−1.58
	(3.75)	(3.68)	(3.46)	(4.23)
GHQ12	0.03	−1.25	0.50	0.22
	(3.78)	(4.26)	(5.61)	(5.06)
POMS	−2.47	0.25	0.41	−0.72
	(9.96)	(10.07)	(8.54)	(10.86)
CES-D	−1.81	-0.39	−0.32	−0.50
	(5.56)	(5.92)	(5.41)	(5.30)

Note: BT: Brain training, AT: active control training, SFN: sulforaphane, P: placebo, POMS: total mood disturbance (TMD) score in Profile of Mood State, WHO-5: World Health Organization-Five Well-Being Index, GHQ12: General Health Questionnaire with 12 items, CES-D: Center for Epidemiologic Studies Depression Scale.

## Data Availability

The datasets used and analyzed in the current study are available from the corresponding author upon reasonable request.

## Supplementary Materials

The following are available online at https://www.mdpi.com/2072-6643/13/2/352/s1, Table S1: CONSORT 2010 checklist of information to include when reporting a randomised trial.
